# The next decade for clinical medical physics

**DOI:** 10.1120/jacmp.v15i6.5334

**Published:** 2014-11-08

**Authors:** Per H. Halvorsen, Michael D. Mills

**Affiliations:** ^1^ Dept. of Radiation Oncology Lahey Health Burlington MA USA

## Abstract

This issue's editorial is an invited commentary authored by Per H. Halvorsen.* It discusses an essential question for clinically practicing medical physicists: How are external factors likely to change the way we practice our profession in the next decade? The topic is both timely and essential, as the AAPM is actively engaged in developing guidance on many related aspects. This editorial sets the framework and provides the personal observations of an individual who has led the AAPM's Professional Council for the past six years.

## INTRODUCTION

*“Because 2024 will not look anything like 2014.”* A local college near my home in the Boston area has adopted this statement as their motto — focusing on their new degree program in big data analytics and how “prescriptive analytics” is poised to reshape health‐care delivery in the US. Perhaps we should adopt it as our motto as well, because I believe we are poised to undergo a significant shift in how we practice clinical medical physics, largely due to external factors that are unavoidable and beyond our control. The challenge to our profession is how we choose to respond, by being willing to assess and reconsider the things we can control.

### The external factors

*The American Recovery and Reinvestment Act of 2009* ^(1)^ included a section crucial to the health‐care sector, called the HITECH Act. In addition to the Meaningful Use incentives, the Act includes seed funding for electronic exchange of health information and for interoperable clinical data repositories. The subsequent years have produced the desired results (whether those effects are truly causal or not) — significant research and development by commercial firms and academic institutions into big‐data analytics in health care. Applications are likely to move from predictive analytics to prescriptive analytics — that is, using analytics tools and decision algorithms to make decisions rather than merely to inform decisions. Tied to this are the Medicare Clinical Quality Measures and Physician Quality Reporting System,^(2)^ whereby incentives are tied to clinical quality metrics intended to discourage overutilization of expensive services and better coordination of care. Some current incentives focus on avoiding overutilization of bone scans for staging low‐risk prostate cancer and avoiding the overuse of cardiac stress imaging. The initial stages utilized payment incentives (additional reimbursement for those who reported on the metrics), but starting in 2015 the approach changes from carrot to stick — reimbursement rates will be reduced by 2% to 5% over the next five years for failure to comply.

*The Patient Protection and Affordable Care Act of 2010* ^(3)^ (“ObamaCare”) includes a shift toward primary care with a resultant pressure on reimbursement and/or utilization of specialty services. The Act includes pilot programs for episodic payments, with an expanded definition of an “episode of care” that includes the full continuum of care, from hospitals and physician services to skilled nursing, rehabilitation, and home health services. Pilot programs are already in operation and are likely to lead to expanded implementation of delivery models such as Medical Homes and Accountable Care Organizations.

At the same time, the Centers for Medicare and Medicaid Services (CMS) is moving aggressively toward expanded use of bundling in the existing payment systems, the Outpatient Prospective Payment System (OPPS) and Medicare Physician Fee Schedule. The existing Ambulatory Payment Classification (APC) groups have recently been expanded with the introduction of the Comprehensive‐APC concept. From CMS' own Web site, *“CMS created Comprehensive‐APCs to prospectively pay under the OPPS for high cost device dependent services in 29 device dependent APCs using a single payment for the hospital stay.”* ^(4)^ Note the emphasis on high cost device dependent services — that, my friends, defines our specialties.

So the overall trend from these external factors is a move toward population‐based health management with emphasis on primary care (at the expense of specialty care) and a new payment scheme that moves away from valuation of individual procedures and instead focuses on the average cost of caring for a given diagnosis across a large patient population. In this context, our specialties will move from being considered revenue centers to being considered cost centers.

### Useful precedents and enabling technologies

I recently attended the ACR's Radiology Leadership Institute 2014 Summit, where the faculty and attendees spent considerable time discussing the radiologist profession's recent evolution and how the radiologists are responding. This may be a useful precedent for our own specialty. Over the past decade, the radiologists have faced significant pressure from the introduction of teleradiology and the trend toward other specialists acquiring their own imaging systems and even interpreting their own images. These dual trends were leading toward a commoditization of the radiologist profession. After significant debate and “soul searching”, the profession responded by accepting the need for more cost‐effective delivery through Radiologist Assistants and the ACR Appropriateness Criteria, while also focusing on providing more professional value and visibility to caregivers outside radiology — for example, bringing evening and weekend call back under the local radiologist practice, participating in multidisciplinary rounds, and serving on hospital committees implementing EHRs and new data‐driven operational models (radiologists are well practiced in data‐driven models). Over the past decade, the nature of the radiologist's role has evolved; they've had to crack some previous “sacred cows” and individual members have recognized the need to contribute a non‐negligible amount toward safeguarding the profession's future, but the profession remains healthy and well respected today. I believe we can learn from their experience, and we should be prepared to address our own “sacred cows” and comfortable habits.

We should also be prepared to fully leverage the benefits of improved tools and well‐defined quality control (QC) procedures to provide routine QC services in a more cost‐effective manner, while enabling Qualified Medical Physicists (QMP) to provide their services to a broader segment of the health‐care system. The medical physics profession, particularly through the AAPM, has provided very good guidance on appropriate QC schemas for each imaging and therapy modality, and the broad adoption of these recommendations has produced a significant experience base. At the same time, the accelerator and imaging‐system manufacturers, as well as many well‐respected third‐party manufacturers, have developed improved instruments and software suites to simplify the routine data collection for these QC schemas. As a profession, we must be willing to consider new paradigms for performing the most common routine QC work, including the use of Medical Physicist Assistants (MPAs) in a well‐defined framework that addresses the appropriate range of tasks, supervision levels, and training requirements for MPAs.

### Path toward a new model for delivering clinical medical physics services

If we're not willing to transform the practice of clinical medical physics over the next decade, our services may become commoditized and our profession marginalized.

A successful transformation for the clinical medical physics profession requires that the QMP become a more visible consultant and resource to health systems in safety assessments and QC program design, as well as becoming a competent manager of other technical employees, rather than limiting our scope to the familiar range of QC tests and clinical procedure support. To do this, we need to improve our management and leadership skills, and we need to learn to operate at a health system level, rather than a department level. This, in turn, requires that we develop the bandwidth to engage at this new level — and that means being willing to re‐examine what we do today to determine what can be safely delegated to others with appropriate supervision, and what can be safely eliminated due to improved manufacturing tolerances and/ or improved monitoring tools.

The first step in this transformation is to accept the need to transform, and the second step is to develop the willingness to re‐examine and re‐tool how we perform our current‐state scope of services. The third step is to develop our management and leadership skills, and to learn how to identify opportunities to engage at the health system level in addition to our individual departments.

I hope you'll join me in taking these first steps toward a transformed clinical medical physics profession. With our joint efforts, I'm optimistic that 2024 will show our specialty to be in a healthy state with a bright future ahead for new entrants into this wonderful profession.

## ACKNOWLEDGEMENTS

I'd like to thank and acknowledge the following groups of colleagues for constructive discussions and keen insight on the aforementioned topics: The members of the AAPM Professional Council, Professional Economics Committee, and the Strategic Planning Committee of the Board.
